# Distinct cortical activity patterns in Parkinson’s disease and essential tremor during a bimanual tapping task

**DOI:** 10.1186/s12984-020-00670-w

**Published:** 2020-03-17

**Authors:** Frauke Luft, Sarvi Sharifi, Winfred Mugge, Alfred C. Schouten, Lo J. Bour, Anne Fleur van Rootselaar, Peter H. Veltink, Tijtske Heida

**Affiliations:** 1 Department of Biomedical Signals and Systems, Faculty EEMCS, University of Twente, Postbox 217, 7500 AE Enschede, The Netherlands; 2 Department of Neurology, Amsterdam Neuroscience, Amsterdam UMC, University of Amsterdam, Amsterdam, Netherlands; 3 Faculty of Mechanical, Maritime and Materials Engineering, Delft University of Technology, Delft, Netherlands; 4 Department of Biomechanical Engineering, University of Twente, Enschede, The Netherlands

**Keywords:** Parkinson’s disease, Essential tremor, Bimanual tapping, EEG, Accelerometers, Task related power, Cueing

## Abstract

**Background:**

Parkinson’s disease (PD) and essential tremor (ET) are neurodegenerative diseases characterized by movement deficits. Especially in PD, maintaining cyclic movement can be significantly disturbed due to pathological changes in the basal ganglia and the cerebellum. Providing external cues improves timing of these movements in PD and also affects ET. The aim of this study is to determine differences in cortical activation patterns in PD and ET patients during externally and internally cued movements.

**Methods:**

Eleven PD patients, twelve ET patients, OFF tremor suppressing medication, and nineteen age-matched healthy controls (HC) were included and asked to perform a bimanual tapping task at two predefined cue frequencies. The auditory cue, a metronome sound presented at 2 or 4 Hz, was alternately switched on and off every 30 s. Tapping at two different frequencies were used since it is expected that different brain networks are involved at different frequencies as has been shown in previous studies. Cortical activity was recorded using a 64-channel EEG cap. To establish the cortical activation pattern in each group, the task related power (TRP) was calculated for each subject. For inter-groups analysis, EEG electrodes for divided into 5 different areas.

**Results:**

Inter-group analysis revealed significant differences in areas responsible for motor planning, organization and regulation and involved in initiation, maintenance, coordination and planning of complex sequences of movements. Within the area of the primary motor cortex the ET group showed a significantly lower TRP than the HC group. In the area responsible for combining somatosensory, auditory and visual information both patient groups had a higher TRP than the HC group.

**Conclusions:**

Different neurological networks are involved during cued and non-cued movements in ET, PD and HC. Distinct cortical activation patterns were revealed using task related power calculations. Different activation patterns were revealed during the 2 and 4 Hz tapping task indicating different strategies to execute movements at these rates. The results suggest that a including a cued/non-cued tapping task during clinical decision making could be a valuable tool in an objective diagnostic protocol.

## Background

The basal ganglia and the cerebellum are brain structures involved in the preparation, timing and execution of timed movements. In movement disorders, such as Parkinson’s disease (PD) and Essential tremor (ET), these structures can be affected. PD is considered a disease of the basal ganglia, and ET a disorder related to changes in the cerebellum [1]. Despite their pathological differences, PD and ET can be difficult to distinguish from each other, due to overlapping symptoms. Common diagnostic tools, such as polymyography, movement disorder rating scales or SPECT scans are either invasive (SPECT), time consuming, subjective (rating scales), expensive and/or not widely available.

Several studies have investigated movement parameters and cortical and subcortical changes in patients and healthy controls during hand and finger movements. In a previous study [2] we showed that PD patients tap significantly less accurately during a 2 Hz tapping task and with a greater variability during a 4 Hz tapping task than ET patients and healthy controls (HC). Furthermore, ET patients tapped less accurately and with a greater variability than HC during a 4 Hz tapping task. Findings were similar for cued and non-cued conditions. Most interestingly the occurrence of kinetic tremor during a tapping task seemed to not affect or even improve the performance of PD patients, but seemed to decrease performance in ET. Gerloff et al. [3] showed that different cortical activation patterns can be recorded in healthy subjects during internally and externally cued finger movements using task-related power calculations of two frequency bands: part of the alpha (9–11 Hz) and part of the beta band (20–22 Hz). These bands have previously been shown to be sensitive to movement-related changes in cortical activity [3]. Changes in activation pattern were found in the supplementary motor areas, primary sensory motor area and lateral premotor cortex during internally timed and externally cued movements. The mesial frontocentral cortex and the ipsilateral sensorimotor cortex were primarily activated during internally cued movements. Another frequency band that is altered in PD in the gamma band [4]. Activity in the gamma band is known to represent engaged networks and facilitating movement [4].

Samuel et al. [5] found evidence that in PD patients the parietal-lateral premotor circuits are activated instead of the striato-mesial frontal circuits to facilitate complex finger movements. A functional MRI study [6] showed that PD patients exhibited an increased activation in the cerebellum and the frontostriatal circuit during externally cued movements and a greater involvement of the cerebello-thalamic circuit compared to HC during internally timed movements.

In ET patients, a study has shown impairment of rhythm generation and increased variability of rhythmic hand movements during cued movements [7]. Furthermore, Avanzino et al. [8] found that 1 Hz-rTMS over the ipsilateral cerebellum affected the performance during a finger movement task in patients with ET, by reducing touch duration values and normalizing the inter touch interval values.

However, comparing the cortical activation patterns of PD and ET patients during cueing has not yet been done, let alone used in clinical decision making. Therefore, the aim of this study is to determine if there are differences in cortical activation patterns in PD and ET patients and HC during cued and non-cued movements. For this purpose, all participating subjects performed a bimanual tapping task at two frequencies, i.e. they tapped with both hands simultaneously and in synchrony with the cue frequency. The subjects were asked to continue movement at approximately the same pace after the cue was turned off. It is hypothesized that PD patients show increased activation of the lateral cortical areas instead of the striato-mesial frontal area during non-cued, internally timed movements due to increased activity in the parietal-lateral premotor circuits as a compensatory strategy using the cerebello-thalamic circuit [5]. In ET patients, reduced activation of the motor cortex is expected during cued movements as hyperactivity of the cerebellum increases the inhibitory output of the thalamus to the motor cortex.

## Methods

### Participants

Eleven PD patients (3 Female (F); Age: 66 ± 11 (mean ± std. (years)), 12 ET patients (4 F; Age: 58 ±20) and 19 age-matched HC (9 F; Age: 59 ± 15) were included in the study. Gender was not considered to be of influence on the results. Therefore, due to a higher incidence rate in men than in women in both patient groups [9–11], more males were included than females, but the HC group was not gender matched. See Table 1 for an overview of patient details. Tremor rating scores and tremor severity are not included in this table as both, occurrence and severity, can be very task dependent. Instead the tremor presence per task will be calculated. All subjects were right-handed according to the Edinburgh Handedness Inventory [12] and patients were willing to stop tremor suppressing medication according to a personalized scheme, established by an experienced neurologist, prior to the experiment. The scheme was based on half-life time of the medication and all patients were assessed in ON- and OFF-state. Additionally, PD patients were diagnosed with Parkinson’s disease according to the UK Brain Bank criteria for Parkinson’s disease [13] and showed neither major fluctuations in symptoms due to medication nor suffered from severe dyskinesia. ET patients had essential tremor according to the criteria defined by the Tremor Investigation Group [14], expressed moderate to severe tremor (Tremor Rating Scale Part A2 UE > 2) and had a positive family history of ET. Healthy subjects had no record of a neurological or other disorder. All subjects gave written informed consent prior to participation and the study was approved by the Medical Ethical Committee of the Academic Medical Center, Amsterdam, The Netherlands.

**Table 1:** Overview of patient details

Subject	Sex	Age	Duration (years)	Medication
ET	M	50	Since birth	–
ET	F	81	21	–
ET	M	85	Unknown	Propranolol
ET	F	51	Childhood	–
ET	F	23	Childhood	–
ET	M	49	9	Propranolol
ET	M	54	38	–
ET	M	70	Unknown	–
ET	M	64	Unknown	–
ET	M	55	Childhood	–
ET	M	27	Birth	–
ET	F	81	61	–
PD	M	69	5	Azilect, MAO-Bi, Propranolol
PD	M	67	4	Atane
PD	F	81	5	Sinemet, Metopropol
PD	F	62	2	Levodopa
PD	M	71	2	–
PD	F	43	3	Arane, Requip
PD	M	68	14	
PD	M	64	10	Sinemet, CR d., madopar
PD	M	67	8	Duodopa, symmetrel, stalvo, euthyrox
PD	F	56	5	Levodopa, ropinirol
PD	M	64	7	–

*PD* Parkinson’s disease, *ET* Essential tremor, *M* Malem, *F* Female

### Experimental setup

**Fig. 1:**
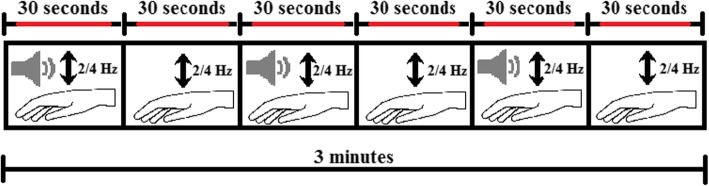
Schematic presentation of the tapping task. Schematic presentation of the bimanual tapping task. 30 s of cued movements was followed by 30 s of non-cued movements. An auditory cue was used, a metronome sound presented at either 2 or 4 Hz. This was repeated 3 times in total and at two and four Hz as cue frequencies. The red line indicates the data segment used for analysis from each block

Subjects were seated on a bed, with head and back supported for a sitting posture. Prior to the tapping task resting state EEG was recorded for 3 min. Afterwards, subjects performed a bimanual wrist flexion tapping task at two cue frequencies, 2 and 4 Hz (equivalent to an inter-tap interval of 500 ms and 250 ms, respectively). The cue was a metronome sound set to the predefined cue frequencies. The forearm and proximal part of the wrist joint were supported against gravity by the bed. Subjects had to continuously tap with both hands positioned on the bed next to their legs in-phase with the cue for 3 min at each cue frequency. The 3 min were split into blocks of 30 s, with alternately the auditory cueing switched on and off. Instructions were given verbally prior to the experiment and all subjects were able to perform the task without practice. During cued movements, subjects were able to hear a metronome beat through computer speakers. Prior to the experiment, subjects were asked whether they were able to clearly hear the sound. The three-minutes tapping task was first performed at 2 Hz and then at 4 Hz, with several minutes rest in between. A schematic overview of the task is given in Fig. 1. In addition to the tapping task, subjects performed a three-minutes resting task with hands resting comfortably in their lap, palms up.

Hand movement was recorded with 3D accelerometers (TMSi, Oldenzaal, The Netherlands – see Introduction for specifications). Muscle activity was recorded from the m. extensor carpi ulnaris of both arms using surface electromyography (EMG) electrodes. EEG was recorded with a 72-channel Refa-system and a 64-channel low-noise shielded EEG head cap (TMSi, Oldenzaal, The Netherlands) (resistance < 30 kΩ). Data acquisition was done using a customized program written in LabVIEW (National Instruments, Austin, Texas, United States) with a sampling frequency of 2048 Hz.

### Data pre-processing

To minimize transition effects (from cued to non-cued movement and vice versa), the first and last 5 s of each block were excluded from analysis (see Fig. 1). Voluntary movement was analysed using the norm of the accelerometer vectors, giving one acceleration signal for each hand. The acceleration signal for each hand was filtered off-line (non-causal, zero-phase, 0.25–20 Hz bandpass, 4th order Butterworth). EMG data was filtered off-line (non-causal, zero-phase, 20–400 Hz, 4th order Butterworth). Then the absolute value of the Hilbert transform was used as the envelope of the EMG signal for further analysis.

**Fig. 2:**
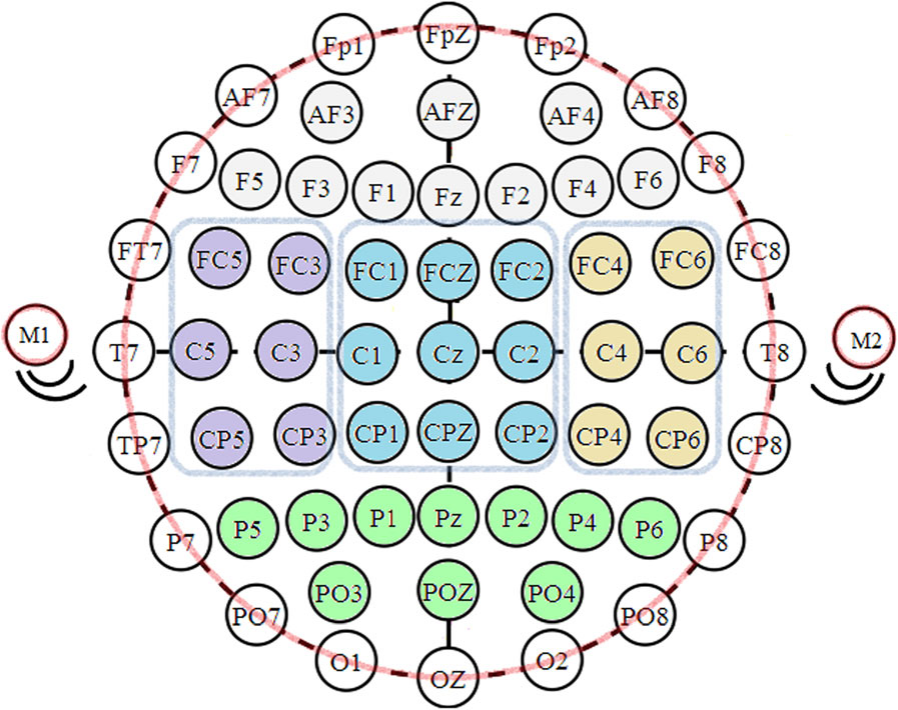
Border electrodes and areas for statistical analysis. Overview of the EEG electrodes. Red regions indicate the border electrodes which were excluded during analysis. Blue and non-circled regions indicate the 5 defined areas for statistical analysis. Grey = frontal area; purple = central left area; beige = central right area; blue = central area and green = posterior area

The second minute of the resting state EEG data, recorded prior to the tapping tasks, was used for further analysis and analyzed using the same pre-processing steps as describe below for the tapping task. The EEG data recorded during the tapping tasks was split into cued and non-cued movement segments. Each segment of the tapping task and the resting state was filtered digitally off-line with a band-pass filter (non-causal, zero-phase, 1–45 Hz; 4th order Butterworth). Eye movement artefacts were removed prior to further analysis using independent component analysis. To minimize reference and volume conduction effects at distances of approximately the inter-electrode spacing [15], a local average reference was used after artefact removal. The montage is described by eq. 1.

1 $$ {V}_{ref,i}={V}_i-\frac{1}{N}\sum \limits_{j=1}^N{V}_{ij} $$

with *V*_*i*_ is the potential at electrode i, *Vi*_*j*_ are the potentials of the neighbouring electrodes and N (*N* = 8) the number of neighbouring electrodes for non-border electrodes. Border electrodes were excluded from analysis as they often contain EMG artefacts and have fewer neighboring electrodes to calculate the local average, leaving 42 electrodes for analysis (see Fig. 2). Furthermore, the mean and the standard deviation of the power of the entire time signal (time signal squared = P) was calculated. Afterward the data was divided into 500 ms epochs with 50% overlap. Only epochs with a mean power, *P*_*epoch*_, according to eq. 2 were included:

2 $$ mean\left({P}_{epoch}\right)< mean(P)+3\ast std(P) $$

### Data analysis

The presence of kinetic tremor during each task was calculated using the EMG data and eq. (3) [2].

3 $$ \mathrm{Kinetic}\ \mathrm{Tremor}:\frac{\sum \limits_{\mathrm{f}=5}^{\mathrm{f}<14}\mathrm{Pxx}\left(\mathrm{f}\right)/{\mathrm{N}}_{\mathrm{TF}}}{\sum \limits_{\mathrm{f}=1}^{\mathrm{f}<5}\mathrm{Pxx}\left(\mathrm{f}\right)/{\mathrm{N}}_{\mathrm{MF}}}\ge 0.8 $$

With Pxx(f) the power at each frequency, f, NTF the number of samples in the tremor frequency band and NMF the number of samples in the movement band (1–5 Hz). After preprocessing, the mean power at each electrode in the frequency domain was calculated for frequencies, up to 45 Hz, using the power spectral density (PSD). The PSD of all epochs was averaged per subject and per task.

### Outcome parameters

The percentage of tremor presence during each task was determined for each subject by calculating the percentage of epochs in which kinetic tremor was detected. From the accelerometer data the tapping accuracy [2] was calculated for each task and tapping condition according to (4).

4 $$ {\mathrm{TA}}_{\mathrm{Subject}}=\frac{\sum \limits_{\mathrm{n}=1}^{\mathrm{N}}\left({\mathrm{f}}_{\mathrm{cue}}-{\mathrm{f}}_{\mathrm{tap},\kern0.5em \mathrm{n}}\right)}{\mathrm{N}} $$

The task related power (TRP) was calculated according to eq. 5 for cued and non-cued movement.

5 $$ {TRP}_{x,i}=\frac{P_{task_{x,i}}-{P}_{rest_{x,i}}}{\max \left({P}_{rest_{x,i}}\right)} $$

For each of the following bands, x, the TRP was calculated for each electrode, i, 9–11 Hz, 20–22 Hz and 30–45 Hz. TRP < 0 indicates a desynchronization, a decrease in activity, during movement compared to rest and TRP > 0 indicates synchronization, an increase in activity, compared to rest.

### Statistical analysis

Statistical analysis was performed to determine differences between groups. Differences in tapping accuracy was determined using multivariate ANOVA analysis. To determine differences in cortical activity EEG electrodes were grouped into 5 groups (see Fig. 2): frontal, left central, central, right central and posterior. TRP in these areas was compared between groups (inter-groups analysis) and within groups (intra-groups analysis) using the non-parametric Kruskal-Wallis test due to varying standard deviation and post-hoc analysis using Bonferroni correction for multiple comparison. A *p*-value smaller than 0.05 was considered significant.

## Results

### Tapping accuracy and percentage of tremor occurrence

The percentage of tremor per task and subject are given in Table 2.

**Table 2:** Tremor presence during tasks

Subject	C2 (%)	NC2 (%)	C4 (%)	NC4 (%)
ET	1	0	0	0
ET	5	82	6	21
ET	0	0	0	0
ET	27	52	7	12
ET	31	0	3	0
ET	0	0	0	17
ET	99	90	19	12
ET	0	1	0	0
ET	0	18	0	0
ET	37	78	17	13
ET	8	0	12	0
ET	21	37	0	0
PD	2	86	20	91
PD	0	0	46	0
PD	2	0	10	0
PD	0	0	0	0
PD	6	15	0	0
PD	0	0	0	0
PD	0	2	0	6
PD	0	0	0	0
PD	3	0	0	0
PD	2	7	0	0
PD	0	0	0	0

*PD* Parkinson’s disease, *ET* Essential tremor, *C2* Cued tapping task at 2 Hz, *NC2* Non-cued tapping task at 2 Hz, *C4* Cued tapping task at 4 Hz, *NC4* Non-cued tapping task at 4 Hz

**Fig. 3:**
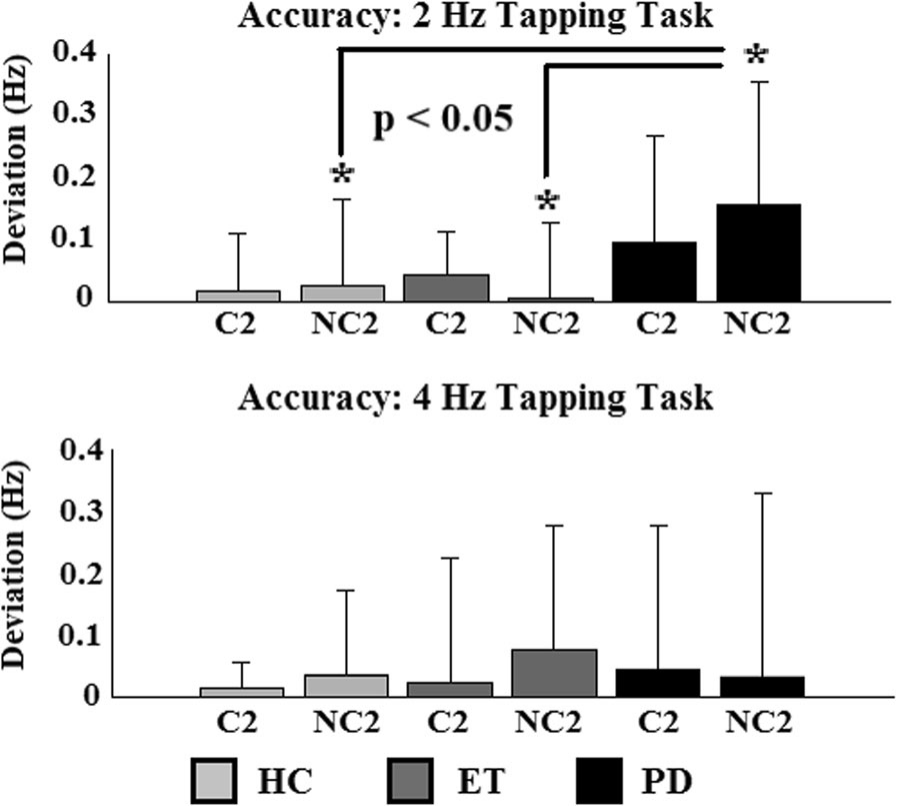
Tapping accuracy. Group results of the tapping accuracy during the 2 and 4 Hz tapping task with and without cue. Statistical differences are marked by an asterisk. HC = Healthy controls; ET = Essential tremor; PD = Parkinson’s disease; C2 = cued tapping task at 2 Hz; NC2 = non-cued tapping task at 2 Hz; C4 = cued tapping task at 4 Hz; NC4 = non-cued tapping task at 4 Hz

In Fig. 3 the results of the tapping accuracy are given. Significant differences were only found during the non-cued 2 Hz tapping task (NC2), indicated by the asterisk. PD patients tapped significantly less accurate than the other two groups.

### Task related power

**Fig. 4:**
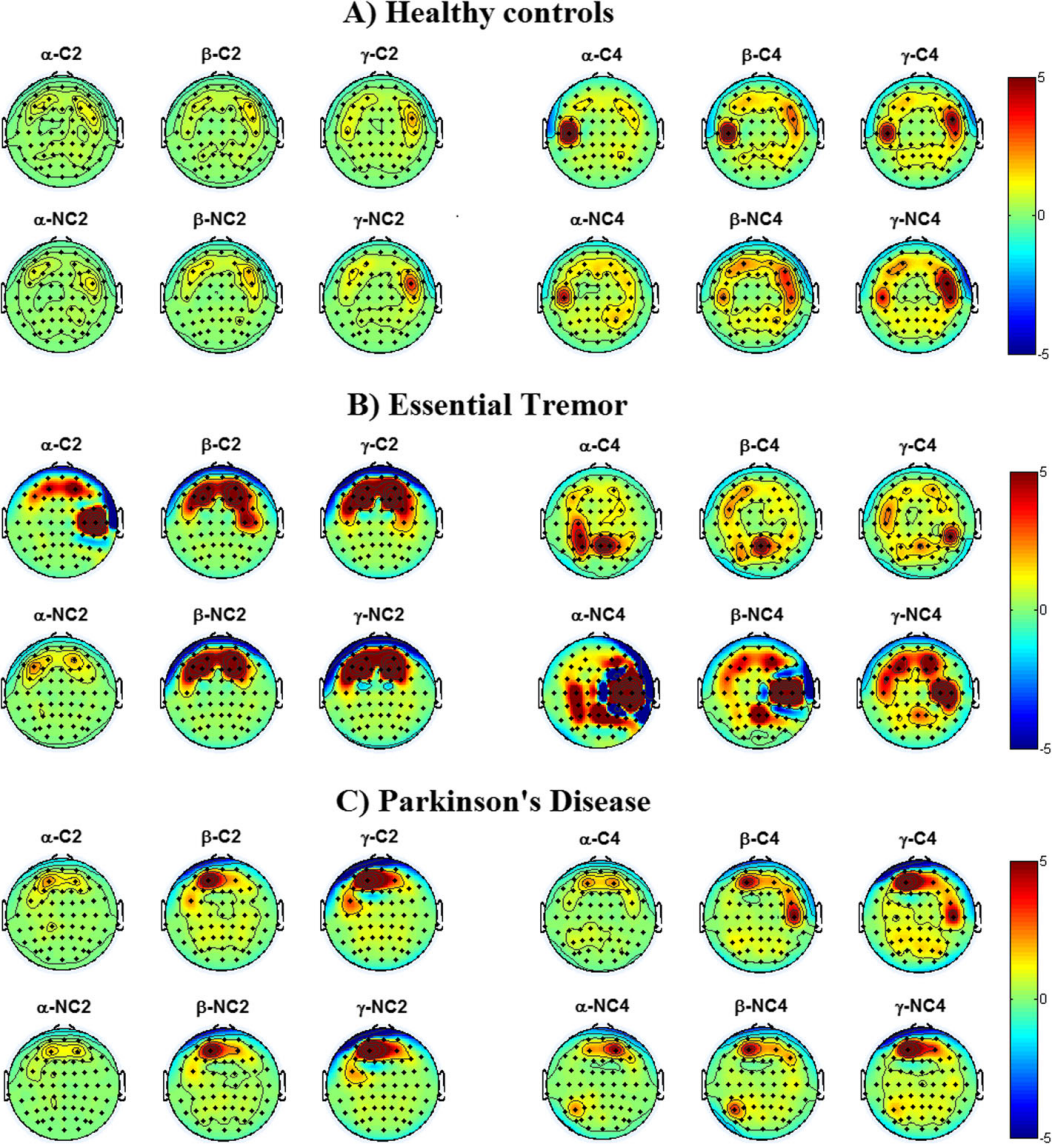
Mean task related power of the three groups. Mean group results of the HC (**a**), ET (**b**) and PD (**c**) group. TRP in the narrow alpha, narrow beta and gamma band during the two tapping tasks with both tapping conditions. Green areas indicate areas with no changes in power between the task and rest; yellow and red indicate an increase in power during the tapping task and blue indicates a decrease in power during the tapping task compared to rest. C2 = cued tapping task at 2 Hz; NC2 = non-cued tapping task at 2 Hz; C4 = cued tapping task at 4 Hz; NC4 = non-cued tapping task at 4 Hz

In Fig. 4 the results of the TRP per task and frequency band are given in topoplots for the HC (A), ET (B) and PD (C) group, respectively. Results represent the mean group result.

### Intra-groups analysis

#### Healthy controls

In the HC group (Fig. 4a), during the 2 Hz cued task (C2), the alpha and beta band show a slightly positive TRP in the dorsolateral prefrontal, intermediate frontal, middle frontal cortex and the pars opercularis. In the gamma band a positive TRP is found around the pars opercularis, premotor and posterior transverse temporal cortex. The 2 Hz non-cued task activity patterns were similar with a slightly more positive TRP seen in the gamma band at the pars opercularis. During the 4 Hz tapping task with cue (C4) an increase in TRP is again seen in the posterior transverse temporal cortex in all frequency bands. In the beta and, even more so, gamma band, additional activity is found around the pars opercularis. Cortical activity recorded during the non-cued 4 Hz tapping task (NC4) shows a similar pattern compared to the C4 task, but with a higher TRP, especially around the anterior transverse temporal cortex in the beta and gamma band. No significant intra-group differences were found.

#### Essential tremor

In the ET group (Fig. 4b) a positive TRP in all three frequency bands during both tapping tasks was found. During the C2 and NC2 task, the frontal areas around dorsolateral prefrontal, intermediate frontal and the premotor cortex are activated in the beta and gamma band. In the alpha band during the C2 task additional activation of the primary auditory cortex was found in the ET group. The C4 task results in a positive TRP around the primary somatosensory cortex, angular gyrus and somatosensory association cortex in the alpha and beta band. In the gamma band activation of the supramarginal gyrus is seen. During the NC4 task activation of the somatosensory association cortex is also found in all three frequency band. Additional activation in all three bands is found around the primary somatosensory and anterior transverse temporal cortex. Furthermore, activation of the dorsolateral prefrontal, intermediate frontal and the premotor cortex is found in the beta and gamma band, but to a lesser extend compared to the C2 and NC2 task. Intra-group differences were only found between the C2 and NC2 task in the alpha band in the frontal area and in the gamma band in the posterior area, with a higher TRP during the C2 task.

#### Parkinson’s disease

In the PD group (Fig. 4c) activity patterns in the C2 and NC2 are almost the same. The alpha band shows slight activation of the dorsolateral prefrontal cortex. In the beta and gamma band activation of the dorsolateral prefrontal cortex increases compared to the alpha band contralateral to the dominant hand. Additional activation of the premotor cortex is found in both frequency bands. Also, during the C4 task activation of the dorsolateral prefrontal cortex is found. Furthermore, activation of the primary auditory cortex is found in the beta and gamma band. During the NC4 task similar activation of the dorsolateral prefrontal cortex is seen. Additionally, activation of the angular gyrus is found, highest in the beta band. The only significant intra-groups difference was found in the posterior area between C4 and NC4 in the alpha band, with a higher TRP during the NC4 task.

### Inter-groups analysis

The results of the inter-group analysis are displayed in Table 3. In the frontal, central and posterior areas significant differences were found most often. Furthermore, the NC4 task resulted in the largest differences between the two patient groups and the HC group. However, significantly different results were not found between the two patient groups.

**Table 3:** Inter-group differences

		Frequency band		
Task	Area	alpha	beta	gamma
C2	Frontal	–	–	–
	Central left	–	0.004^c^	–
	Central	–	0.019^c^	–
	Central right	–	–	–
	Posterior	< 0.001^a^	0.015^c^	< 0.001^d^
NC2	Frontal	–	–	–
	Central left	–	0.039^c^	–
	Central	0.013^b^	–	–
	Central right	–	–	–
	Posterior	< 0.001^a^	–	–
C4	Frontal	< 0.001^a^	< 0.001^a^	< 0.001^a^
	Central left	–	–	–
	Central	0.012^b^	–	–
	Central right	–	–	–
	Posterior	< 0.001^a^	0.003^c^	< 0.001^d^
NC4	Frontal	< 0.001^a^	< 0.001^a^	< 0.001^a^
	Central left	–	–	–
	Central	0.017^b^	–	–
	Central right	–	–	–
	Posterior	< 0.001^b^	0.003^c^	< 0.001^d^

Superscripts indicate which groups show significant differences. ^a^ HC higher TRP than ET and PD; ^b^ HC higher than ET; ^c^ ET higher TRP than HC; ^d^PD and ET higher TRP than HC; *C2* Cued tapping task at 2 Hz, *NC2* Non-cued tapping task at 2 Hz, *C4* Cued tapping task at 4 Hz, *NC4* Non-cued tapping task at 4 Hz

## Discussion

PD and ET are degenerative neurological disorders involving different parts of the brain. The overlapping symptom, tremor, and identifying differences in tremor characteristics has been the focus of many studies [16–19]. The overall goal of this study was to determine different cortical activation patterns during a bimanual tapping task at 2 and 4 Hz, performed with and without an auditory cue. Significantly different activation patterns were found between the HC group compared to both patient groups and between the HC group and the ET group. Even though, no statistically significant differences were found between the two patient groups the results suggest that different activity patterns are involved during cued and non-cued movements in ET and PD and HC.

### Tapping accuracy and percentage of tremor occurrence

The tapping accuracy was calculated to determine the ability of the subjects to perform the task correctly. The only significant difference between the groups was found during the NC2 task indicating that under all other conditions all subjects were able to perform the task in a comparable manner. It also shows that PD patients benefit from an auditory cue, as to being able to perform the task more accurately with cue than without an external cue.

Kinetic tremor was detected in both groups. The duration of the tremor during a task varied per subject and task. Overall, kinetic tremor was recorded more often and for a longer period of time in the ET group compared to the PD group. This was to be expected as ET is characterized by a postural and/or kinetic tremor. However, we also see a clear task dependency of tremor occurrence in both groups.

### Task related power

Significant differences in activation patterns were found mainly in the frontal, central and posterior area.

### Intra-groups analysis

#### Healthy controls

The frontal area is mainly responsible for motor planning, organization and regulation and is involved in initiation, maintenance, coordination and planning of complex sequences of movements. In most cases the HC group had a significantly higher TRP in this region indicating that these areas are important for a high tapping accuracy [2]. In the posterior area, areas responsible for combining somatosensory, auditory and visual information and also areas that are involved in motor learning and bimanual manipulation are located. In the gamma band both patient groups had a higher TRP than the HC group. This might indicate that PD and ET patients both have to make use of more than one peripheral feedback mechanism to initiate and maintain a sequential movement. In the HC group a positive TRP was found in the area of motor planning, organization and regulation during the C2 and NC2 task in the alpha and beta band. Furthermore, areas involved in initiation, continuation and coordination of movements were active. In the gamma band areas responsible for selective attention to rhythm and processing auditory stimuli were active. During the C4 and NC4 task additional activations were found in the areas of motor response inhibition and bilateral object manipulation. Contralateral to the dominant hand activation of areas responsible for movement organization was found. A study by Walsh et al. [20] suggests that the dominant hemisphere initiates the activity responsible for bimanual movement. Likewise, we only found an increase of the primary motor cortex on the contralateral side of the dominant hand during the 4 Hz tapping task.

Other areas were found in the patient groups indicating pathological or compensatory activation of cortical areas in order to perform the desired task.

#### Essential tremor

In the ET group an increased TRP was found in areas involved in motor planning, movement initiation, maintenance, coordination and planning of complex sequences during the C2 and NC2 task. These findings suggest that, in contrast to HC, patients with ET need to plan and monitor simple bilateral hand movements more closely than HC to be able to execute them correctly. The primary motor cortex, areas responsible for selective attention to rhythm and the somatosensory association cortex lay within the central region. This area showed a significantly lower TRP in the ET group than the HC group in the alpha band during the NC2, C4 and NC4 task. This is in accordance with our hypothesis that the hyperactivity of the cerebellum results in less activation of the motor cortex. Furthermore, in the C4 task areas responsible for combining somatosensory, auditory and visual information were activated, indicating that ET subjects need more feedback from the periphery to maintain rhythmic movements. That visual information is important in cerebellar disorders has been shown in other studies as well [21]. In the NC4 task additional activation of areas involved in movement organization, learning motor sequences and the control of rhythmic motor tasks was found, which is in contrast to our hypothesis. It could be that ET patients need more control of rhythmic motor tasks compared to HC. As we only record cortical activity we are not able to determine which underlying activity causes the increased primary motor cortex activity we recorded. This will be investigated in a future study.

#### Parkinson’s disease

In the PD in the beta and gamma bands a positive TRP was found in areas responsible for motor planning, organization and regulation, contralateral to the dominant hand during the C2 and NC2 task. Activation of the frontal areas might indicate a compensatory mechanism of PD to perform a movement task by relying more on motor planning compared to HC. During the C4 task a positive TRP is seen in areas responsible for motor response inhibition, auditory priming and basic processing of auditory stimuli. As in the ET group activation of the area involved in combining somatosensory, visual and auditory information was activated in the NC4 task indicating that PD patients need more peripheral feedback to perform the task than HC.

### Inter-groups analysis

That no differences in TRP were found between the groups could have several reasons. First of all the group size was rather small. Furthermore, tremor occurred in several subjects in both patient groups (Table 2). It could be that kinetic tremor in ET and in PD have at least some common pathways resulting in similar cortical activity during these tremor episodes. In addition to this, freezing or hastening could have occurred in some subjects adding to the heterogeneity within the groups.

### Used methodology

In this study the TRP was calculated using the difference in cortical activity of each subject with respect to his or her own resting activity. Other studies showed that differences in resting activity between HC and PD [22] and HC and ET [23] exist. These differences in activity during rest could also be part of the findings we showed in this study. From literature it is known that PD and ET subjects show pathological changes in cortical resting activity [23–25] compared to HC. This could also have led to the lack in activation of the lateral motor areas in PD with our method as these might already have been pathologically activated during rest. However, the increase in TRP found in this study was related to movement, auditory or sensory processing which is to be expected when executing a movement task under different auditory conditions. Therefore, we conclude that the determined changes are task related and not based on differences in underlying resting activity.

Another limitation is the use of frequencies equal or larger than 2 Hz. Stegemöller et al. [26] showed that the tapping ability in PD patients was not significantly different from HC below 2 Hz. The idea behind this phenomenon is that below 2 Hz the cerebellum is mainly involved in movement control and above 2 Hz the basal ganglia. Therefore, using frequencies below 2 Hz could result in even greater differences between PD and ET and it is suggested to use frequencies below and above 2 Hz in future studies. Furthermore, this could also explain why we did not find increased lateral activation in the PD group and why we did not find significant differences between the patient groups.

## Conclusion

The results of this study show significant differences in activation patterns during a bimanual tapping task in patients with PD and ET compared to a group of HC. Using TRP did not reveal the exact pathological networks involved in movement generation in ET and PD, but revealed distinct activation patterns during a bimanual tapping task. In contrast to our hypothesis, primarily the frontal regions were activated during the tapping task in PD. Therefore, we reject our hypothesis concerning PD. Furthermore, reduced activation of the motor cortex was found in the ET group compared to the HC group. Therefore, the hypothesis concerning reduced activation of the motor cortex due to hyperactivity of the cerebellum is confirmed. Even though, no significant differences were found between the two groups the results suggest that including EEG recording in combination with the performance of a simple tapping task during clinical decision making could be a valuable tool that is not exploited by the scaling indices currently used to diagnose patients and score disease severity. The beta band during the C2 task and the alpha band in the NC4 task showed significant differences between ET and HC but not HC and PD. Therefore, an activation pattern similar to the HC group during clinical decision making is a more likely indicator for PD than ET. Therefore, including a cued/non-cued tapping task seems to provide a promising tool to an objective diagnostic protocol.

## Data Availability

The datasets used and/or analyzed during the current study are available from the corresponding author on reasonable request.

## Abbreviations

C: Central
C2: Bimanual tapping task with cue at 2 Hz
C4: Bimanual tapping task with cue at 4 Hz
ET: Essential Tremor
F: Frontal
HC: Healthy controls
NC2: Bimanual tapping task without cue at 2 Hz
NC4: Bimanual tapping task without cue at 4 Hz
P: Posterior
PSD: Power spectral density
TRP: Task related power
